# Reflection Rumination Reduces Negative Emotional Processing During Goal-Directed Behavior: An ERP Study †

**DOI:** 10.3390/bs15081081

**Published:** 2025-08-08

**Authors:** Max Owens, Jessica Renaud, Ashly S. Healy

**Affiliations:** 1 Department of Psychology, University of South Florida Saint Petersburg Campus, 140 7th Ave S, St. Petersburg, FL 33701, USA; 2 Department of Psychological Sciences, Texas Tech University, 2700 18th St, Lubbock, TX 79406, USA; ashlheal@ttu.edu

**Keywords:** rumination, event-related potential, cognitive control, negative bias

## Abstract

Trait rumination is a repetitive and often maladaptive attentional focus on the consequences of depression. Rumination independently contributes to cognitive control dysfunction associated with depression. However, it is not clear how the effects of rumination on cognitive control may contribute to negative attention biases as well, or whether it is specific to brooding or reflective rumination. To address these questions, the current study examined the link between trait rumination, cognitive control, and attentional biases. Participants were given a task to remember three neutral faces across a delay period with a single irrelevant sad, happy, or scrambled face distractor. Memory accuracy was also collected. Additionally, the amplitude of the emotion processing late positive potential (LPP) component was recorded by electroencephalograph (EEG) in response to distractors. Brooding and reflection were not associated with memory accuracy. Brooding was not significantly related to LPP amplitudes. A significant emotion by reflection interaction on LPP amplitudes was observed. As the reflection levels increased, the LPP amplitudes for sad faces decreased relative to amplitudes for scrambled faces. The effects were maintained while controlling for brooding and depression. The results suggest that reflection may bias attention toward control over negative distraction but not improve accuracy and, thus, may contribute to cognitive inefficiency associated with depression.

## 1. Introduction

Depression is a complex brain disorder that significantly affects emotions and cognitive function, contributing to global disability and burden of disease (Proudman et al., 2021; WHO, 2025). Therefore, understanding depression and its influencing factors is crucial, particularly the role of rumination, which is linked to individual differences in depression and is a transdiagnostic factor related to anxiety, eating disorders, suicidal ideation, insomnia, and impulsive behaviors (Watkins & Roberts, 2020). Rumination is a style of thought characterized by a repetitive and narrowed attentional focus on the causes and implications of one’s problems (Whitmer & Gotlib, 2013). Trait forms of rumination are often measured by self-report, and there is empirical evidence for at least two subtypes of rumination: brooding and reflection (Treynor et al., 2003). Brooding is often a passive comparison of one’s current state with an unachieved standard (e.g., “Think about a recent situation, wishing it had gone better”). Reflection is often characterized by purposefully turning inward to engage in problem-solving (e.g., “Write down what you are thinking and analyze it”). Both are positively correlated with depression concurrently, but brooding predicts increased duration of episodes (Treynor et al., 2003). Thus, understanding how rumination affects emotion processing and cognitive function is important for understanding its underlying mechanisms and how it may then contribute to risk for depression and related disorders.

Traditionally, research on emotion processing and cognitive function in depression has separately focused on the negative biases and concentration difficulties that characterize vulnerability to the disorder (Villalobos et al., 2021). Attention bias research shows that depressed individuals struggle to disengage from negative content in working memory (WM), especially with longer stimulus presentations (i.e., ≥1500 ms; Koster et al., 2005). Rumination has been linked to this biased and sustained processing, impairing attention control, particularly toward negative stimuli (Joormann & Tanovic, 2015; Koster et al., 2011). Evidence from eye movement studies has provided some support for this view, showing that trait forms of rumination are positively associated with negative biases and reduced attention to positive stimuli during passive viewing (Duque et al., 2014; Owens et al., 2016). Although this pattern is not always observed, for instance, Quigley et al. (2024) found that attentional biases were not associated with levels of rumination independently of depression. In contrast, recent meta-analyses demonstrate that greater rumination affects cognitive performance, reducing the ability to suppress the effect of distracting and irrelevant information on behavior (Yang et al., 2017; Zetsche et al., 2018). This aligns with the attention bias literature, suggesting that rumination contributes to difficulties inhibiting irrelevant information in WM. Furthermore, the adverse effects of rumination on cognitive performance often occur independently of mood and stimulus valence (Whitmer & Gotlib, 2013).

Although cognitive control and attentional biases associated with depression are usually examined separately, it has been suggested that they interact through their connections to rumination (Villalobos et al., 2021). This proposal highlights that rumination may affect an individual’s motivation to decide when and how cognitive control is applied and, thus, impact behavior in depression (Grahek et al., 2019). These views are consistent with the conceptualization of rumination as a style of thought (Whitmer & Gotlib, 2013). However, it is not clear how the effects of rumination on cognitive control may contribute to negative attention biases or whether it is specific to brooding or reflective rumination. Therefore, exploring the relationship between rumination, cognitive control, and attentional biases together may help clarify its role in dysregulated behavior and its effects on depression in general. So, the primary aim of the current study is to examine how levels of rumination influence sustained attention to irrelevant emotional and neutral information in WM during goal-directed behavior.

Emotional stimuli can impair cognitive control, increasing distractibility during goal-directed behavior (Dolcos & Denkova, 2016). Research often uses delayed response WM paradigms to examine cognition–emotion interactions (Wong et al., 2012). Neuroimaging studies show that emotional distraction reduces activity in prefrontal cognitive control regions while increasing activity in emotion processing regions like the ventrolateral prefrontal cortex and amygdala. The effect of emotional distraction resembles the neural correlates of maladaptive forms of rumination (Zhou et al., 2020). In contrast, trait levels of reflection rumination tend to load less on these regions and have been associated with increased activity in regions associated with cognitive control within MDD patients (Hamilton et al., 2011; Jacob et al., 2020).

Additionally, electrophysiological studies have provided some evidence that trait levels of brooding and reflection relate to different styles of information processing during goal-directed behavior. In electrocochleogram (EEG) studies, this effect of rumination on information processing is often reflected as an altered pattern of event-related potential (ERP) amplitude levels (Alderman et al., 2015; Krompinger & Simons, 2011; Palmwood et al., 2017). On a verbal Stroop color-naming task, high levels of rumination in non-clinically depressed individuals have been associated with increased N450 congruency effects, which reflect stimulus conflict detection (Krompinger & Simons, 2011). A related amplitude pattern has been observed in MDD patients using the flanker task, with levels of trait rumination associated with reduced N2 amplitudes on incongruent flanker trials, indicating an impaired response to conflict detection (Alderman et al., 2015). Finally, in a cued Go/NoGo Stop-Signal task, trait levels of reflection rumination across MDD patients and controls were associated with reduced differences in P300 amplitudes between successful and unsuccessful Stop-Signal behavior, indexing the implementation of inhibition in the task (Palmwood et al., 2017). Overall, electrophysiological evidence highlights the consistent effects of rumination on neurocognitive function in non-clinical and patient samples, lending support to its role in risk for depression. Additionally, often levels of rumination in these studies were not significantly associated with task performance. So, it has been argued that rumination contributes to inefficient neurocognitive function. In this view, the pattern of altered neural activity observed in rumination during goal-directed behavior reflects increased task effort that does not translate to improved behavioral outcomes (Krompinger & Simons, 2011; Palmwood et al., 2017).

This study investigated the interaction between cognitive control and sustained emotion processing using an electrophysiological delayed-match-to-sample WM task (Wong et al., 2012). In the current study, the late positive potential (LPP) was measured to examine the level of sustained attention to neutral, happy, and sad face distractors. There is evidence that trait rumination is positively associated with LPP amplitudes for affective words (Lo et al., 2012) in response to losses versus gains in a gambling task (Webb et al., 2017) and for self-referential compared to non-self-related processing (Hsu et al., 2021). Furthermore, using LPPs to index emotion processing (Gan et al., 2019), it was observed that rumination mediates the relationship between reduced cognitive control for negative faces and levels of depression. These findings are consistent with evidence that has linked LPP to emotion regulation and the allocation of attention to the motivational salience of stimuli (MacNamara et al., 2022). Overall, the literature suggests that the LPP can effectively measure how rumination affects sustained attention to irrelevant emotional and neutral stimuli. However, to our knowledge, no study has examined the relationship between brooding, reflection, and emotion processing in a goal-directed task using the LPP. We hypothesized that levels of brooding rumination would be positively associated with biased processing of emotional distractors, leading to increased LPPs during the delay period for negative faces and reduced accurate memory performance for targets [i.e., neutral faces]. In contrast, reflection may be expected to increase cognitive control and reduce LPPs during the delay period. Finally, if rumination affects the efficiency of cognitive control, lower LPP amplitudes and no association between rumination and task performance would be expected (Krompinger & Simons, 2011; Palmwood et al., 2017).

## 2. Materials and Methods

### 2.1. Participants

This study was advertised online through the University of South Florida St. Petersburg campus automated experiment management system. A total of 46 participants took part in this study and were reimbursed for their time with $10.00 or $20.00, depending on the level of course credit received. From this pool, 6 participants were excluded from the analyses, 4 participants were completely missing questionnaire data, 1 participant had missing behavioral data, and 1 participant was excluded from the analyses due to a BDI score greater than 3 standard deviations from the sample mean. The participants included for the analyses (*n* = 40) ranged in age from 19 to 38 years old (*M* = 25.87, *SD* = 5.88). A total of 75% of the participants in the study were female, and 92% were right-handed. All study procedures were approved by the Institutional Review Board of the University of South Florida.

### 2.2. Measures

#### 2.2.1. Rumination

The short-form Ruminative Response Scale (RRS; Treynor et al., 2003) was used to assess levels of trait rumination. The short-form RRS is a 10-item self-report questionnaire assessing the frequency that ruminative thoughts or behaviors occur when the person is feeling sad, down, or depressed. Scores on the scale range from 1, Almost Never, to 4, Almost Always, and are summed for all items, totaling 40, with higher scores indicating an increased tendency toward rumination. For the current study, we used the two five-item subscales that assess rumination subtypes: brooding (*M* = 9.38, *SD* = 3.34, range = 5–20) and reflection (*M* = 9.89, *SD* = 3.54, range = 5–18). Consistent with previous research, good internal consistency was observed for all 10 items (α = 0.87), brooding (α = 0.82), and reflection (α = 0.78). The rumination subcomponents were significantly correlated with each other and depression levels in the current sample: reflection and brooding, *r*(38) = 0.52, *p* < 0.001; reflection and depression, *r*(38) = 0.36, *p* = 0.01; and brooding and depression, *r*(38) = 0.70, *p* < 0.001. Additionally, sex was not significantly correlated with levels of depression, *r*(38) = 0.03, *p* = 0.85, brooding, *r*(38) = −0.02, *p* = 0.89, and reflection, *r*(38) = −0.06, *p* = 0.70, in the current study.

#### 2.2.2. Depression Severity

Depression, as well as other forms of psychopathology, may negatively affect individuals from low to high levels rather than primarily along categorical dimensions (Cuthbert & Kozak, 2013). In line with this view, the current study focused primarily on continuous symptom levels, as measured by self-report. Levels of depression symptoms were assessed with the Beck Depression Inventory II (Beck et al., 1996). The inventory consists of 21 items assessing the severity of symptoms of depression. Each item has a four-point scale ranging from zero to three. The total scores reflect the severity of depressive symptoms. Minimal levels of depression range from 0 to 13, mild is 14–19, moderate is 20–28, and severe is total scores between 29 and 63. In the current study, the participants’ levels of depressive symptoms were in the minimal to severe range (*M* = 10.20, *SD* = 9.68; range = 0–36). The BDI-II exhibited excellent internal consistency in this sample (α = 0.94).

#### 2.2.3. Emotional Delayed Response WM Task and Stimuli

**Figure 1:**
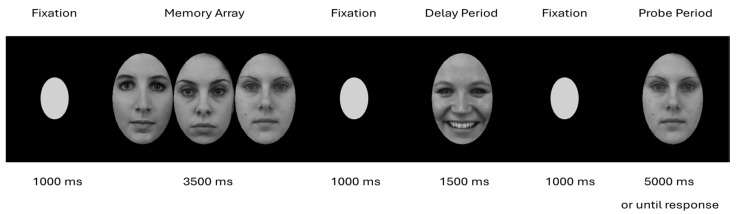
Example of a happy distractor condition in a match trial. The participants were instructed to remember the identities of the neutral faces in the memory array across a delay period and ignore face images in the delay period. During the probe period, the participants’ memory of the neutral faces was tested with a single neutral face probe that remained on screen for 5000 ms or until the participants’ responses.

The participants performed an emotional delayed response WM task adapted from Wong et al. (2012). Figure 1 shows an example trial in the task. Each trial started with a central fixation lasting 1000 ms. The participants were then presented with a memory array with three neutral faces for 3500 ms. The participants were instructed to remember the identities of the faces across a delay period. Before the delay period, a central fixation was presented for 1000 ms. During the delay period, a single sad, happy, or scrambled face image could appear for 1500 ms, and the participants were instructed to ignore the faces or images in the delay period and focus on remembering the neutral faces. In the current study, we used scrambled face images for the neutral distractor stimuli, as neutral faces can elicit emotional responses in depression (Leppänen et al., 2004), and the LPP is sensitive to this arousal as well (Weinberg & Hajcak, 2010). After the delay period, the participants were presented with a central fixation for 1000 ms, while a brief tone indicated the upcoming probe period. During the probe period, the participants’ memory of the neutral faces was tested with a single neutral face probe that remained on screen for 5000 ms or until the participants’ responses. The participants responded with a button press indicating whether the probe face was a previously seen old face from the memory or a new face in the trial. The participants completed a short practice of four trials with neutral distractor images before completing 72 trials divided evenly across all distractor and memory probe types. The task stimuli consisted of 48 neutral faces used as targets only and 26 sad faces, 26 happy faces, and 26 neutral faces divided evenly across male and female faces and drawn from a combination of Karolinska Directed Emotional Faces (Lundqvist et al., 1998), NimStim (Tottenham et al., 2009), and the Chicago Face Database (Ma et al., 2015) stimulus sets. Using MATLAB (R2019b), the faces were trimmed of all non-facial features, converted to 8-bit grayscale, and matched for luminance and contrast. The greyscale neutral facial stimuli were scrambled using MATLAB (R2019b).

### 2.3. EEG Recording and Processing

During the task, continuous EEG was recorded using a BrainVision actiCHamp 64 (Brain Vision LLC, Morrisville, NC, USA) channel active electrode system using a fitted cap. The horizontal electrooculogram (EOG) was recorded from two electrodes placed 1 cm to the left and right of the external canthi to measure horizontal eye movements. A vertical EOG was recorded from a single electrode placed below the left eye to measure eye blinks. The electrode impedance was kept below 10 kΩ. EEG data were recorded in reference to the right mastoid. The EEG recordings were filtered online with a bandpass at 0.01–80 Hz and sampled at 1000 Hz. An offline analysis was performed using the MATLAB extension EEGLAB (Delorme & Makeig, 2004) and the EEGLAB plug-in ERPLAB (Lopez-Calderon & Luck, 2014). The EEG data were first down-sampled to 250 Hz and then re-referenced to the average of the left and right mastoid electrodes and filtered with cutoffs of 0.1 Hz and 30 Hz. The EEG data were processed using both artifact rejection and correction. The large and stereotypical ocular components were identified and removed using an independent component analysis (ICA) of scalp maps (Jung et al., 2000). Epochs with large artifacts (greater than 100 μV) and blinks at the distractor presentation were excluded from the analysis. The EEG data were segmented for each trial, beginning 200 ms before the onset of the delay period and ending 1500 ms after the onset of the delay period. The period of 200 ms before each trial served as a baseline for the ERP analyses. Only correctly answered trials were used for the analyses (Happy, *M* = 14, *SD* = 4; Sad, *M* = 15, *SD* = 4; Neutral, *M* = 15, *SD* = 4). The number of correct trials for each distractor condition was unrelated to depression, brooding, and reflection, lowest *p* = 0.45.

**Figure 2:**
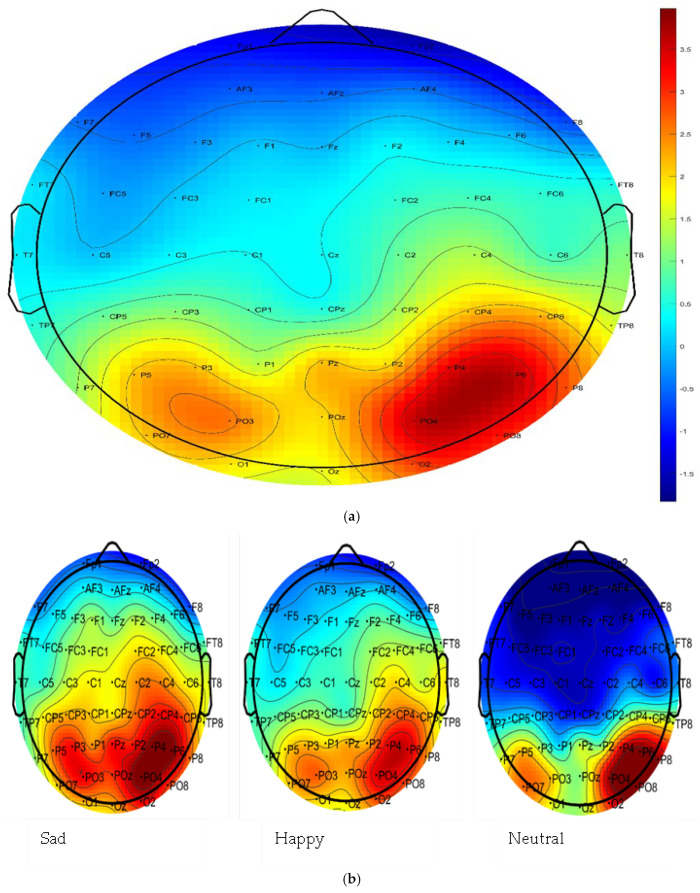
Grand-averaged scalp maps during the delay period (0–1500 ms). (**a**) Mean delay period amplitudes averaged across region (left, midline, and right), distractor gender (male and female), distractor emotion (sad, happy, and neutral), and trial type (match and no match). (**b**) Mean delay period amplitudes for each distractor type averaged across hemisphere (left, midline, and right), distractor gender (male and female), and trial type (match and no match).

We used a collapsed localizer approach to compute the LPP (Luck & Gaspelin, 2017). Firstly, grand-averaged ERP data were collapsed across distractor type and visually inspected to determine the areas of greatest regional activity for the LPP (Figure 2). Consistent with the literature (Weinberg et al., 2021), scalp maps showing the highest amplitudes for the LPP were located within the central and parietal regions. Within the parietal regions, peak activity is apparent at two midline electrodes (Pz and POz) and left (P4, P6, P8, PO4, PO8, and O2) and right (P3, P5, P7, PO3, PO7, and O1) lateralized electrode sites.

**Figure 3:**
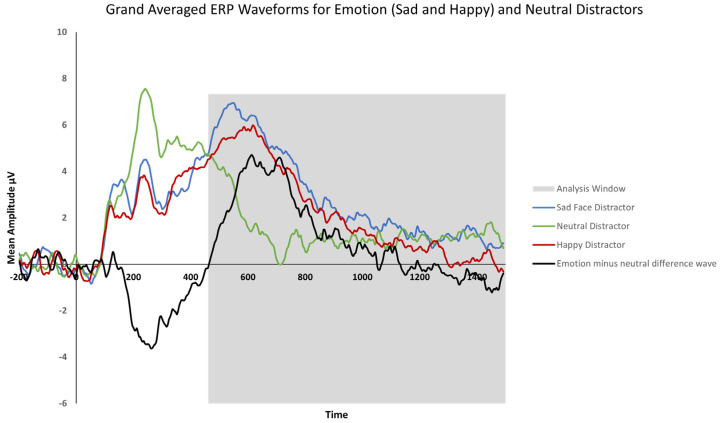
Grand-averaged LPP waveforms for the sad face, happy face, and neutral distractors across the delay period (0–1500 ms). The waveforms were averaged across the midline (Pz, POz), right (P4, P6, P8, PO4, PO8, and O2), and left (P3, P5, P7, PO3, PO7, and O1) electrode sites. The difference between emotion and neutral distractor waveforms is shown as well (black line). The highlighted regions show the measurement window (460–1500 ms) for the mean LPP amplitudes used in the analyses.

Figure 3 shows grand-averaged LPP waveforms for the neutral distractor, sad and happy face distractors, the average of emotional distractors, and an emotion minus the neutral difference wave across the parietal midline, left, and right hemispheres. Typically, the LPP begins approximately 400 ms after the stimulus presentation and is characterized by increased amplitudes for emotional compared to neutral stimuli (Hajcak & Foti, 2020; MacNamara et al., 2012). The difference between the neutral and emotion distractor face waveforms is also shown in Figure 3. In line with the literature, the late positive potential (LPP) is apparent within the delay period and shows a more positive amplitude for emotional compared to neutral distractors in the task. Also, in Figure 3, the LPP can be seen as a positive waveform in the emotion minus neutral difference waves that begins approximately 460 ms from the stimulus presentation until the end of the stimulus presentation period (1500 ms). For the analyses, the LPP for the correct trials was computed separately for each electrode (midline, Pz, POz; right, P4, P6, P8, PO4, PO8, and O2; left, P3, P5, P7, PO3, PO7, and O1), distractor (happy, sad, and neutral), distractor gender (female and male), and trial type (match and no match). ERPs for each trial type and stimulus gender were then averaged together for each distractor type by midline, left, and right hemisphere regions.

## 3. Results

### 3.1. Behavioral Data

The average accuracy by distractor type was happy trials (*M* = 75%, *SD* = 0.12), sad trials (70%, *SD* = 0.14), and neutral trials (75%, *SD* = 0.11). In line with the expectations, higher LPP amplitudes, averaged across all electrode regions, were associated with lower accuracy overall in the task, *r* = −0.37, *p* = 0.01, [−0.61, −0.07]. The levels of depression and rumination (brooding and reflection) were not correlated with accuracy in the task; all *p*’s > 0.1.

### 3.2. ERP Data

#### 3.2.1. Analysis Steps

The data were analyzed using IBM SPSS Statistics (version 29). First, the effects of emotion and region on mean LPP amplitudes were analyzed in a repeated-measures ANOVA with emotion (happy, sad, and neutral) and region (midline, left, and right) as the within-subjects variables. Continuous levels of brooding or reflective rumination were then added as between-subjects variables in separate general linear models. The difference from a repeated-measures ANOVA is that general linear models allowed us to keep brooding and reflection as continuous variables rather than artificially dichotomizing them, which could lead to Type I or Type II errors (MacCallum et al., 2002). The results of these analyses are reported in Table 1. For each analysis, Greenhouse–Geisser-corrected values are reported when appropriate.

#### 3.2.2. Emotion and Region Effects

As shown in Table 1, a significant interaction between emotion and region was observed and, thus, was first probed for each emotion by region. For happy faces, *F*(2,78) = 3.68, *p* = 0.049, no pairwise comparison reached significance, left electrode sites (*M* = 1.85, *SD* = 2.78) compared to right electrode sites (*M* = 2.64, *SD* = 3.39), *p* = 0.054, and all other comparisons with midline electrode sites (*M* = 2.15, *SD* = 3.25), *p*’s > 0.2. For sad faces, *F*(2,78) = 3.96, *p* = 0.03, the LPP amplitudes at the left electrode sites (*M* = 2.38, *SD* = 2.64) were not significantly different compared to the right electrode sites (*M* = 3.19, *SD* = 3.12), *p* = 0.06, and were significantly lower compared to the midline electrode sites (*M* = 3.14, *SD* = 3.48), *p* = 0.002; the amplitudes for right and midline sites were not significantly different, *p* > 0.1. For neutral distractors, *F*(2,78) = 28.29, *p* < 0.001, the LPP amplitudes at the left electrode sites (*M* = 1.05, *SD* = 2.35) were significantly lower than those for the right electrode sites (*M* = 2.66, *SD* = 2.87), *p* < 0.001, and higher than the midline electrode sites (*M* = 0.52, *SD* = 2.81), *p* = 0.009; the amplitudes for the right and midline sites were significantly different, *p* < 0.001. Next, the interaction was probed for each region by emotion. For the left electrode sites, *F*(2,78) = 3.77, *p* = 0.02, no pairwise comparison reached significance, sad faces compared to neutral face distractors, *p = 0*.051, and all other comparisons, *p*’s > 0.2. For the right electrode sites, *F* < 1, there were no significant differences between the distractor types. For the midline electrodes, *F*(2,78) = 10.78, *p* < 0.001, the mean LPP amplitudes for sad and happy faces were significantly higher than the neutral distractor, and the LPPs for sad and happy faces were not significantly different, *p* > 0.2. Overall, the emotion-by-region interaction shows relatively higher mean LPP amplitudes for sad faces at midline regions compared to the left electrode sites and for both emotional distractors in comparison to the neutral distractors at the midline regions. Additionally, the LPPs for the neutral distractors tended to be lower at the midline regions relative to the lateralized regions. The LPP is often observed to be maximal for emotion compared to neutral stimuli at the midline and parietal regions (Hajcak & Foti, 2020; Weinberg et al., 2021), so the pattern of effects in the current task is in line with the topography of the LPP and its proposed role as an index of the motivational salience of emotion compared to neutral stimuli (MacNamara et al., 2022).

#### 3.2.3. Brooding and Reflection Analyses

As shown in Table 1, there were no significant main effects or interactions with levels of brooding rumination. For levels of reflection, the significant main effects of emotion and reflection were qualified by a significant reflection-by-emotion interaction.

**Figure 4:**
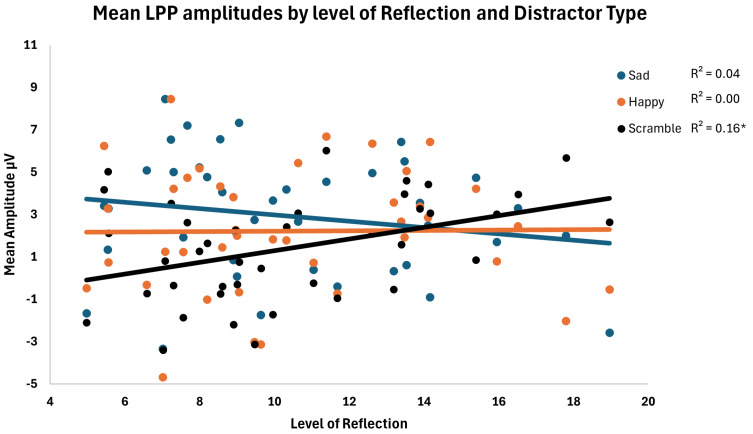
Mean LPP amplitudes by level of reflection and distractor. Regression lines and R-square values for LPP amplitudes by level of reflection rumination for each distractor type are shown as well. * *p* < 0.05.

Figure 4 shows the relationship between the continuous reflection scores and each distractor type. A negative relationship for levels of reflection and LPP amplitudes for sad and happy distractors was observed, but only the positive relationship between neutral distractors was significant. However, across levels of the distractor type, the slope of the relationship between emotion and neutral distractors appears different for levels of rumination. To examine these relationships, difference scores were computed for sad and happy distractor trials minus neutral trials. Additionally, the amplitude difference between sad and happy distractors was computed as well.

The correlation between each difference score and level of reflection rumination was then computed. For the sad distractor trials, as the reflection levels increased, the LPP amplitudes for sad faces significantly decreased relative to the amplitudes for neutral distractors, *r* = −0.45, *p* = 0.003, CI [−0.67, −0.16]. For happy distractor trials, as the reflection levels increased, the LPP amplitudes for happy faces significantly decreased relative to the amplitudes for neutral distractors, *r* = −0.34, *p* = 0.03, CI [−0.46, −0.02]. Finally, the slope between sad and happy trials was not significantly different, *r* = −0.17, *p* = 0.28, CI [−0.62, 0.14].

To determine the robustness of these effects, we examined the relations between reflection and the difference wave amplitudes while statistically controlling for the influence of brooding and then participants’ current BDI-II scores. In these analyses, reflection rumination was still significantly associated with reduced LPP amplitudes for sad distractor faces relative to neutral distractors when controlling for levels of brooding, *r* = −0.34, *p* = 0.03, and depression, *r* = −0.37, *p* = 0.02. For these analyses, the relationship between reflection and the happy−neutral difference waves was non-significant, with both *p*’s = 0.09, suggesting that this relationship was at least somewhat associated with depression and brooding rumination in our sample.

## 4. Discussion

Cognitive models have proposed that trait rumination is associated with reduced cognitive control over emotion; however, research has often explored the effects of rumination on cognitive control and attention biases separately. So, recent proposals have argued for an integrative approach that more directly models how rumination may link these cognitive processes and influence subsequent behavior (Grahek et al., 2019; Villalobos et al., 2021). Supporting the hypothesized link between rumination, cognitive control, and attention biases, we found that increased levels of trait reflection rumination were related to significantly reduced LPP amplitudes to sad and happy distractors relative to neutral distractors in the task. However, only the relationship between high trait reflection and reduced LPP amplitudes for sad distractors remained significant after controlling for levels of brooding and depression, suggesting an independent mood-congruent bias. Additionally, reflection rumination was unrelated to behavioral performance in the task. The results of the current study agree with conceptualizations of reflection as a relatively adaptive form of rumination that engages cognitive control regions in the brain (Hamilton et al., 2011; Treynor et al., 2003). In line with the results of the current study, there is recent empirical evidence that higher levels of reflection at baseline in adolescents predict fewer switch costs between counting angry and neutral faces in an emotional switching task (Stewart et al., 2019). As such, there is some evidence that reflection rumination may facilitate greater task focus. Although the current study did not directly measure cognitive control, greater task-related focus would be expected to result in reduced LPPs to irrelevant emotional distractors (c.f. Gan et al., 2019). However, this increased task-related focus did not translate to the increased behavioral performance that would be expected. As such, the results indicate an inefficient use of cognitive control resources related to high levels of reflection (Krompinger & Simons, 2011). Finally, a medium effect was observed for the reflection by emotion interaction (Cohen, 1988). Therefore, future studies will be needed to confirm these findings, perhaps with a larger sample and using more trials, to further explore the links between cognitive control, rumination, and depression for distractor processing using the LPP.

### Avenues for Future Research

A key strength of this study was the use of ERPs to explore the effect of rumination on emotional distraction during goal-directed behavior. When the distractor type was examined separately, higher levels of reflection were significantly associated with increased mean LPP amplitudes to neutral images, which alone may be interpreted as an increased attention to these distractors. Rumination has been linked to difficulties removing information from WM after it has captured attention (Koster et al., 2011). However, typically, this attention capture is associated with negative attention biases (Joormann & Gotlib, 2010; Koster et al., 2011). Therefore, why reflection would bias attention toward a neutral distractor over emotional distractors during goal-directed behavior is unclear. In contrast, the LPP is generally higher for emotional compared to neutral stimuli and is interpreted to index the motivational salience of stimuli (MacNamara et al., 2022). In the current study, a similar interaction was observed, with mean LPP amplitudes for sad and happy distractors significantly higher than neutral distractors at midline regions. As a result, the slope analyses of the emotion modulation of the LPP by levels of reflection rumination appear to indicate a biased effort to suppress attention to sad distractors relative to neutral distractors, where perhaps less control was allocated. In the absence of such an analysis, more subtle effects of reflection on behavior may have been missed. Suppression is considered a key feature of rumination (Nolen-Hoeksema et al., 2008). As such, future studies should consider how reflection rumination may dynamically affect the allocation of attention to emotional or motivationally salient distractors during goal-directed behavior. Brooding was not significantly associated with attention biases in the current study. However, increased negative biases with high levels of brooding and depression are often observed in naturalistic viewing paradigms (Duque et al., 2014; Owens & Gibb, 2017; Quigley et al., 2024). Brooding is described as a more passive form of emotion processing (Treynor et al., 2003). Therefore, in the current study, the effects of reflection rather than brooding on behavior may have appeared more pronounced. If different forms of rumination play a significant role in the style of emotion processing during goal-directed behavior, future research should consider additional related factors as well, such as sex and motivation.

The response styles theory of depression was developed to explore how rumination may contribute to sex differences in depression. In the current study, most participants in this study were women, which precluded examining moderation of our findings by sex. Brooding, but not reflective rumination, has been found to mediate the sex difference in depression (Treynor et al., 2003). Therefore, it will be important for future studies to determine if sex differences affect how cognitive control and attention are allocated in the task. Recently, models of depression have emphasized motivation as a key mechanism influencing cognitive control dysregulation in the disorder (Grahek et al., 2019). If rumination generally leads to a more effortful but less efficient use of cognitive resources, it may impact when, how, or for how long control is allocated. In other words, rumination may interact with depression at a motivational level to influence task performance (Grahek et al., 2019). In line with this view, a recent neuroimaging study found that rumination is associated with increased neural activation in frontoparietal regions responsible for inhibitory control in a Go/No-Go Stop-Signal task within depressed individuals but not in controls (Park et al., 2024). Future research with experimental designs that allow exploration of neural activation and the impact of related factors may help clarify how each style of rumination contributes to dysregulated cognitive control as well as attentional biases. Additionally, further integrating the role of rumination within a motivational framework of depression may enhance our understanding of the mechanisms underlying cognitive control dysregulation in the disorder.

## 5. Conclusions

The current study supports a link between rumination and increased task focus and further supports a distinction in the processing style of reflection and brooding. The results suggest that in conditions when processing salient distracting information must be avoided, reflection contributes to decreased attention to distractors. However, the way control is allocated may have negative implications for cognitive efficiency and motivated behavior in depression.

## Figures and Tables

**Table 1:** Repeated-measures ANOVA for within-subjects effects and general linear models for continuous levels of brooding rumination and reflection rumination.

	Source	*DF*	*F*	*p*-Value	Partial η^2^
Within-subjects	Emotion	2.78	4.46	0.01	0.10
	Region	2.78	9.18	0.002	0.19
	Emotion × Region	4.156	20.07	<0.001	0.34
Brooding		1.38	0.000	0.986	0.00
	×Emotion	2.76	2.55	0.086	0.06
	×Region	2.76	0.18	0.74	0.005
	×Emotion × Region	4.152	0.228	0.88	0.006
Reflection		1.38	0.19	0.66	0.005
	×Emotion	2.76	5.14	0.009	0.12
	×Region	2.76	2.13	0.12	0.05
	×Emotion × Region	4.152	1.60	0.19	0.04

## Data Availability

The data presented in this study are available on request from the corresponding author.
